# Fullfield and extrafoveal visual evoked potentials in healthy eyes: reference data for a curved OLED display

**DOI:** 10.1007/s10633-022-09897-5

**Published:** 2022-09-10

**Authors:** Sabine Baumgarten, Tabea Hoberg, Tibor Lohmann, Babac Mazinani, Peter Walter, Antonis Koutsonas

**Affiliations:** Department of Ophthalmology, RWTH Aachen University, Pauwelsstr. 30, 52074 Aachen, Germany

**Keywords:** Electrophysiology, Visual evoked potentials, Fullfield pattern-reversal VEP, Extrafoveal pattern onset–offset VEP

## Abstract

**Purpose:**

Visual evoked potentials (VEP) present an important diagnostic tool in various ophthalmologic and neurologic diseases. Quantitative response data varied among patients but are also dependent on the recording and stimulating equipment. We established VEP reference values for our setting which was recently modified by using a curved OLED display as visual stimulator. Distinction is made between fullfield (FF) and extrafoveal (EF) conduction, and the effect of sex, age and lens status was determined.

**Methods:**

This prospective cross-sectional study included 162 healthy eyes of 162 test persons older than 10 years. A fullfield pattern-reversal visual evoked potential (FF-PR-VEP) with two stimulus sizes (ss) (20.4′ and 1.4°) as well as an extrafoveal pattern onset–offset VEP (EF-P-ON/OFF-VEP) (ss 1.4° and 2.8°) was derived in accordance with the International Society for Clinical Electrophysiology of Vision guidelines. Amplitudes and latencies were recorded, and the mean values as well as standard deviations were calculated. Age- and sex-dependent influences and the difference between phakic and pseudophakic eyes were examined. A subanalysis of EF-P-ON/OFF-VEP and fullfield pattern onset–offset VEP (FF-P-ON/OFF-VEP) was performed. A 55-inch curved OLED display (LG55EC930V, LG Electronics Inc., Seoul, South Korea) was used as visual stimulator.

**Results:**

Mean P100 latency of the FF-PR-VEP was 103.81 ± 7.77 ms (ss 20.4′) and 102.58 ± 7.26 ms (ss 1.4°), and mean C2 latency of the EF-P-ON/OFF-VEP was 102.95 ± 11.84 ms (ss 1.4°) and 113.58 ± 9.87 ms (ss 2.8°). For all stimulation settings (FF-PR-VEP, EF-P-ON/OFF-VEP), a significant effect of age with longer latencies and smaller amplitudes in older subjects and higher amplitudes in women was observed. We saw no significant difference in latency or amplitude between phakic and pseudophakic eyes and between EF-P-ON/OFF-VEP and FF-P-ON/OFF-VEP.

**Conclusions:**

A curved OLED visual stimulator is well suited to obtain VEP response curves with a reasonable interindividual variability. We found significant effects of age and gender in our responses but no effect of the lens status. EF-P-ON/OFF-VEP tends to show smaller amplitudes.

## Introduction

Visual evoked potentials (VEP) present an important, non-invasive diagnostic tool in various ophthalmologic and neurologic diseases. They are used to test the visual conduction pathway from the optic nerve to the visual cortex [1]. They provide information concerning sensory function and the integrity of the visual system [2].

Three stimulus modalities are commonly used: pattern-reversal (PR), pattern onset–offset (P-ON/OFF) and diffuse flash stimulation [3]. The preferred stimulus for many cases is PR because of its relatively low variability of waveform and peak latency intra- and interindividually [4].

Main recorded parameters are latency and amplitude. Latency describes the time from stimulus onset to the largest amplitude of a positive or negative deflection [5], and usually, two main peak-to-trough amplitudes are looked at (N75-P100 and P100-N135).

A normal VEP response to a pattern-reversal stimulus consists of a triple-headed waveform: It begins with a negative deflection (N75), followed by a prominent positive spike (P100) and a later negative deflection (N135) [4]. Here, the P100 is the most consistent and shows least variability compared with the N75 and N135 waves [6].

Three main peaks are also seen in standard P-ON/OFF-VEPs: The positive C1 peak is recorded approximately after 75 ms, the negative C2 approximately after 125 ms followed by the positive C3 peak, approximately appearing after 150 ms [5].

Given the potential impact of laboratory-specific factors, such as background lighting conditions or distance between the subject and the stimulus displays each laboratory has to establish its own reference values using its own stimulus and recording parameters [4, 7]. The resulting data collection of a normal sample for reference values should respect age, sex and interocular asymmetry [5]. Anyhow, in the literature there is clear agreement regarding general trends in the normative values [1, 2, 8–14].

In the past, cathode-ray tube (CRT) monitors have been used as visual stimulators in most electrophysiological laboratories, but they were replaced by liquid crystal displays (LCD) and the recently developed OLED (organic light-emitting diode) screens [15, 16]. The characteristics of the latter have been analyzed, and the results proofed the suitability of their use as visual stimulators to elicit pattern VEPs [16]. The specific material properties of OLED screens make it possible to use curved screens, however, not as good as, e.g., in projection spheres being used for visual field testing, but in a first step it reduces at least partly the geometric distortion of the different distances between the pattern elements in the center and in the periphery of the stimulator screen which might be important to obtain VEPs from the periphery.

Depending on which area of the retina is stimulated, different responses are expected. With the development of the binary m-sequence and its use in multifocal VEPs and ERGs, it became possible to deduce cortical potentials depending on the localization of visual stimulation in the visual field [17]. Masking the central five degrees as performed in our study is expected to evoke different VEP responses. Differing pattern sizes will do so as well as finer patterns (< 15′) are supposed to evoke mainly foveal VEPs, whereas coarser patterns (> 30′) evoke VEPs also via extrafoveal stimulation [18].

The present study was performed in order to determine the normative values in fullfield pattern-reversal VEPs (FF-PR-VEP) and extrafoveal pattern onset–offset VEPs (EF-P-ON/OFF-VEP) in healthy test persons, respecting the influence of sex and age. As an experimental approach we deduced extrafoveal VEPs in PR- as well as P-ON/OFF-VEPs.

## Probands and methods

Included in the analysis of this prospective cross-sectional study were 162 healthy eyes of 162 test persons 10 years or older. In total, 69 were males, and 93 were females. Excluded from the analysis were 32 eyes because of inadequate impedance measuring or very poor quality of measuring curves. Evaluation of the curve quality was handled very strictly as the impedance of every electrode had to be < 5 kΩ and a difference of > 1 kΩ between the electrodes should not be exceeded.

To guarantee balanced age distribution test subjects were assigned to different age groups: group A: 10 to 19 years, group B: 20 to 39 years, group C: 40 to 59 years and group D included all study participants that were 60 years or older. In group D, a subdivision was made distinguishing between phakic and pseudophakic eyes (see Table 1). There were no significant differences between the groups concerning sex (*p* = 0.993 from Pearson’s *χ*^2^ test) and age (*p* = 0.940, see Table 2).

**Table 1:** Test persons’ characteristics (number of test persons, mean age, sex, lens status)

	Group A	Group B	Group C	Group D		
				Phakic	Pseudophakic	Total
Number of test persons	28	44	34	32	24	56
Mean age (years) ± standard deviation	17.5 ± 2.0	26.3 ± 4.5	50.9 ± 5.5	69.7 ± 7.0	74.3 ± 7.8	71.7 ± 7.6
Number of males Percentage (%)	12 42.9	18 40.9	15 44.1	15 46.9	9 37.5	24 42.9
Number of females Percentage (%)	16 57.1	26 59.1	19 55.9	17 53.1	15 62.5	32 57.1

**Table 2:** Mean age (years) and standard deviation (SD) of males versus females for the different age groups: A00 = all phakic males of group A (10–19 years), B00 = all phakic males of group B (20–39 years), B10 = all phakic females of group B (20–39 years), C00 = all phakic males of group C (40–59 years), C10 = all phakic females of group C (40–59 years), D00 = all phakic males of group D (sixty or older), D10 = all phakic females of group D (sixty or older), D01 = all pseudophakic males of group D (sixty or older), D11 = all pseudophakic females of group D (sixty or older)

	Group A00	Group A10	Group B00	Group B10	Group C00	Group C10	Group D00	Group D10	Group D01	Group D11
Mean age	17.8	17.3	26.4	26.3	50.5	51.1	71.9	67.7	75.4	73.6
SD	1.2	2.5	4.1	4.9	6.2	5.0	6.3	7.1	6.8	8.5

All VEP measurements were taken in the electrophysiological laboratory of the Department of Ophthalmology at RWTH Aachen University between January 2019 and August 2020. To minimize investigator-dependent influences all deductions were performed by one specialist (TH).

Inclusion criteria were a visual acuity of better than 0.2 LogMAR per eye, and the test person had to reach the age of ten. Exclusion criteria implied a condition after retinal arterial or venous occlusive disease, any history of retinal detachment, strabismus, any glaucomatous optic nerve damage as well as any kind of optic nerve or retinal damage due to underlying diseases such as arterial hypertension or diabetes mellitus. For safety reasons, persons with epilepsy were excluded.

Test persons were recruited from the patient pool and visitors of the Department of Ophthalmology at RWTH Aachen University, providing the above-mentioned inclusion and exclusion criteria were fulfilled.

The Institutional Ethical Review Board of the RWTH Aachen University approved the study (EK204/18). The described research adhered to the tenets of the Declaration of Helsinki.

### VEP measurements

The procedure of the VEP examination was explained to all subjects, and written informed consent was taken. VEP was recorded with a one-channel montage provided by Roland Consult (Brandenburg an der Havel, Germany). Recordings were taken in a darkened room with a quiet environment.

The test person was positioned one meter in front of the 55-inch (height 68.00 cm, width 122.03 cm) OLED monitor (LG55EC930V, LG Electronics Inc., Seoul, South Korea), so the monitor was presented under a visual angle of 37.6°. The radius of the curvature of the monitor was 5000 mm. The resolution was 1920 × 1080 px. The bit level of representation was 8 bit. Signal input was 60 Hz. Input lag was 39 ms measured with the Leo Bodnar device. The input lag was a preset value and was automatically substracted from the latencies. The CPU used in this study was Intel ®Core™ i5-2500 CPU.

Pupils were not treated by miotic or mydriatic drugs, and the test person was optimally refracted for the viewing distance of the screen and respective age.

After cleaning the skin with colorless skin antiseptic (octeniderm® farblos, Schülke & Mayr GmbH, Norderstedt, Germany) and with the electrode paste II produced by the in-house pharmacy (containing tragacanth 5.0 g, glycerol 85% 8.0 g, distilled water 150.0 g, sodium chlorid 34.0 g, potassium tartrat 2.0 g, sorbic acid 0.1 g, potassium sorbat 0.2 g, pumice stone 25.0 g, sea sand 25.0 g), the EEG scalp gold cup electrodes (GRASS® Cup electrodes LTM, 75 cm cable length, diameter 10 mm, 1,5 mm DIN socket, Grass, Italy) were positioned. As conductive-adhesive paste we used the Ten-20 Conductive Paste (Weaver and Company, Aurora, USA) to ensure stable electrical connection. To fixate the scalp electrodes two elastic bands with associated buttons were utilized. Scalp electrodes were positioned according to the Ten-Twenty-System. The active electrode was placed on the scalp over the visual cortex at Oz, the reference electrode was positioned on the forehead at Fz, and the ground electrode was fixated on the vertex, Cz. Referring to the International Society for Clinical Electrophysiology of Vision (ISCEV) standard, electrode impedances were below five kΩ and the impedance should not differ > one kΩ between the active electrode and the reference electrode.

Occlusion plasters (Piratoplast, Dortmund, Germany) covered the fellow eye during monocular testing. Each test person underwent two measuring sessions per eye. Monocular stimulation was given to both eyes separately. Firstly, the FF-PR-VEP protocol was presented. Here, we used two stimulus sizes (ss): For the large stimulus, we used checks with a width of 1.4 angle degree (1.4°). For the smaller ss, checks width was 20.4 min of arc (20.4′).

**Fig. 1:**
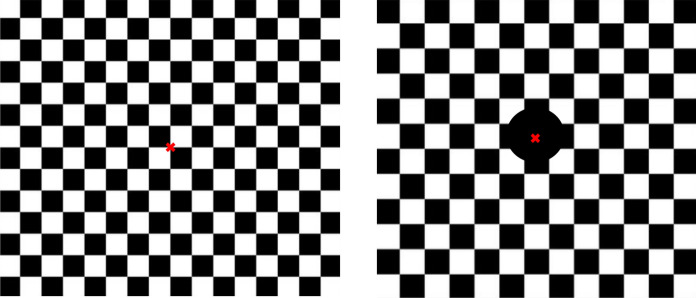
Left: stimulation setting for the fullfield pattern-reversal visual evoked potential (FF-PR-VEP); right: stimulation setting for the extrafoveal pattern onset–offset VEP (EF-P-ON/OFF-VEP) with the central visual field blocked with a black disc with a diameter of 9 cm. In each case, two stimulus sizes (ss) were used (FF-PR-VEP: 1.4° and 20.4′; EF-P-ON/OFF-VEP: 2.8° and 1.4°)

The black and white checks changed abruptly and repeatedly at three reversals per second (1.5 Hertz (Hz)) generating a transient VEP. The analysis time (sweep duration) was 250 ms and more than hundred responses were averaged (number of sweeps). An amplification rate of 20.000 to 50.000 was used. The mean luminance of black checks was 0.58 cd/m^2^ and for the white checks 105.8 cd/m^2^. The parameters of the display were measured by the high-precision luminance meter MAVO-MONITOR (Gossen, Nürnberg, Germany). The contrast between black and white squares was high with 99%, as defined by Michelson contrast. A red fixation cross was positioned in the center at the corner of four checks. The stimulus pattern was presented on the full monitor (see Fig. 1).

Except for slightly larger check sizes and higher reversal rates given above, the recording and stimulus parameters followed the ISCEV Standards for clinical PR-VEPs [19].

In the second measuring program, the EF-P-ON/OFF-VEP was elicited. Here, the central visual field was blocked with a black disc with a diameter of 9 cm placed onto the screens center. Thus, the central 5.15° of the retina was not stimulated. Again, two ss were used. As large stimulus, we used checks with a width of 2.8° and checks with a width of 1.4° for the smaller stimuli. Pattern onset duration was 17 ms and the diffuse gray background appeared afterward for 650 ms. This setting corresponded to the ISCEV-standard from 2004 [4]. Essential was the constant mean luminance of the diffuse background and the checkerboard with no change of luminance during the transition from pattern to diffuse blank screen.

The other recording and stimulus parameters were the same as in the pattern-reversal setting according to the ISCEV standards [19].

In order to compare the EF-P-ON/OFF-VEP with the pattern onset/offset where the central retina is also stimulated (fullfield = FF-P-ON/OFF-VEP), we measured ten test subjects with both investigation programs (EF-P-ON/OFF-VEP vs. FF-P-ON/OFF-VEP).

### Statistical analysis

For descriptive statistics, all metric values were expressed as the mean ± standard deviation (range minimum to maximum). Multivariate linear regression was used for the explorative data analysis (Software IBM® SPSS Statistics, version 25.0). Normal distribution was verified using the Shapiro–Wilk test on a 5% level of significance and considering the graphic distribution by histograms and Q–Q diagrams. If the distribution of the target variable in a subpopulation was not normal, a transformation was performed using the natural logarithm (*y* = ln(*x*)).

Mainly right eyes were evaluated. If there was a test person with one eye pseudophakic and the other eye phakic, we evaluated only one eye per test person for the corresponding group so there was never more than one eye per study subject included. Including both the right and left eye of a single test person was not admitted due to the lower intra-individual variance between right and left eyes of the same subject compared to the variance between subjects [20, 21].

For group D and the analysis of phakic and pseudophakic eyes, an independent samples Student’s *t* test was applied.

For the subanalysis of EF-P-ON/OFF-VEP versus FF-P-ON/OFF-VEP, a paired samples t test was used.

## Results

### Fullfield pattern-reversal VEP (FF-PR-VEP)

**Fig. 2:**
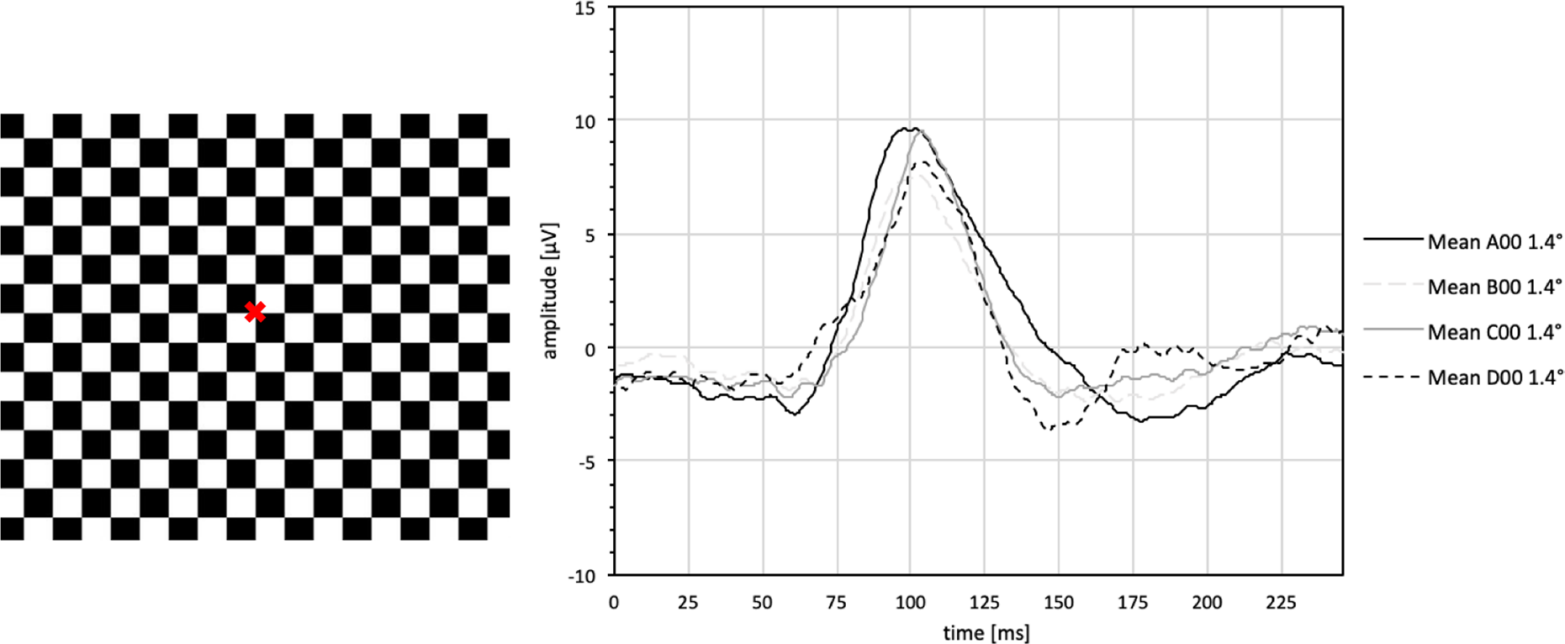
Averaged waveforms of fullfield pattern-reversal visual evoked potential (FF-PR-VEP) of male phakic test persons with 1.4° ss. A00 = all phakic males of group A (10–19 years), B00 = all phakic males of group B (20–39 years), C00 = all phakic males of group C (40–59 years), D00 = all phakic males of group D (sixty years or older)

**Fig. 3:**
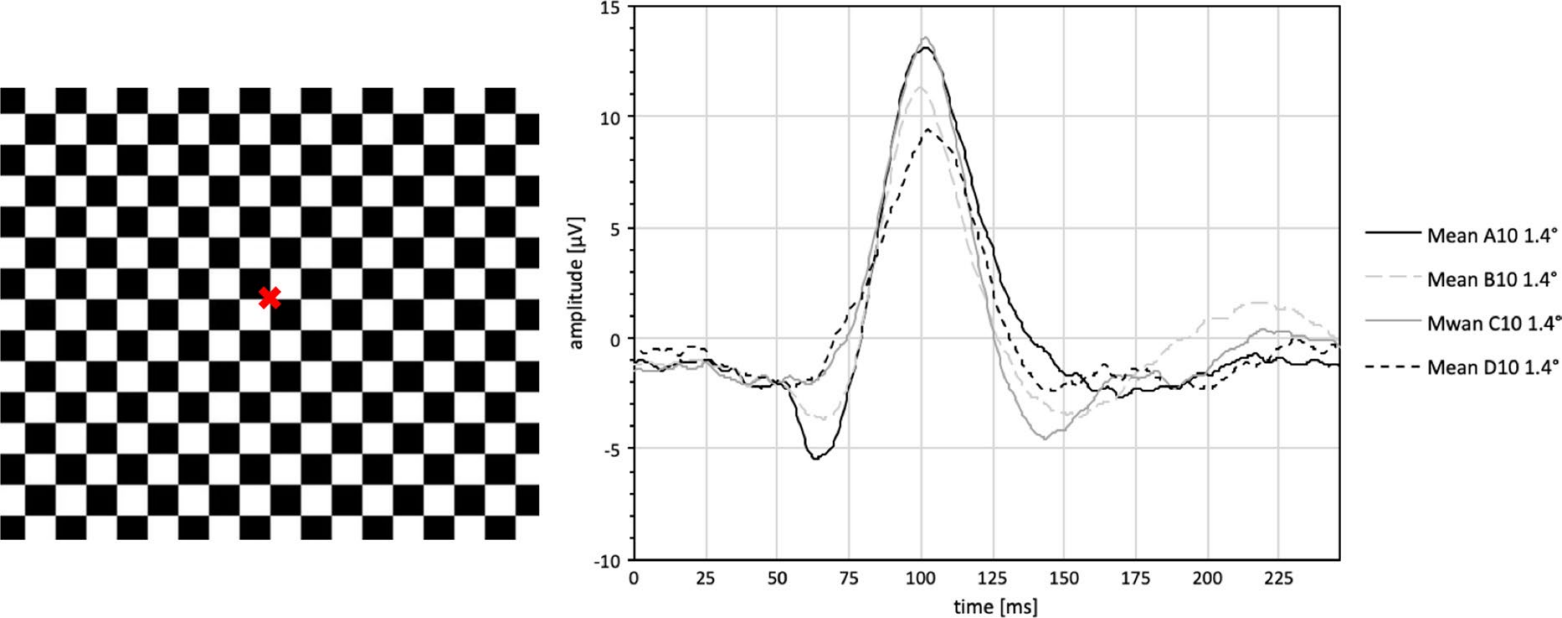
Averaged waveforms of fullfield pattern-reversal visual evoked potential (FF-PR-VEP) of female phakic test persons with 1.4° ss. A10 = all phakic females of group A (10–19 years), B10 = all phakic females of group B (20–39 years), C10 = all phakic females of group C (40–59 years), D10 = all phakic females of group D (sixty years or older)

The VEP as a response to fullfield stimulation (37.6 deg of visual angle) with pattern-reversal checkerboards of 1.4° check size was analyzed for the whole group as well as for each age group of phakic patients (*n* = 138) and is summarized in Table 3, and averaged waveforms are presented in Figs. 2 and 3 for male and female test persons.

**Table 3:** Reference values (mean value ± standard deviation (range)) for latencies (N75, P100, N135) and amplitude (N75-P100) of phakic test persons for the fullfield pattern-reversal VEP (FF-PR-VEP) with 1.4° ss

	N75 [ms]	P100 [ms]	N135 [ms]	N75-P100 [μV]
Total (*n* = 138)	69,55 ± 10,24 (47,20 – 96,50)	102,58 ± 7,26 (85,30 – 125,00)	145,31 ± 15,82 (109,00 – 190,00)	15,66 ± 6,42 (4,40 – 45,30)
Group A (*n* = 28)	67,44 ± 7,00 (50,70 – 80,10)	102,13 ± 8,29 (91,20 – 125,00)	147,68 ± 19,95 (118,00 – 190,00)	18,49 ± 8,26 (6,33 – 45,30)
Group B (*n* = 44)	69,83 ± 9,43 (51,30 – 88,90)	100,01 ± 6,41 (85,30 – 114,00)	147,05 ± 16,78 (114,00 – 189,00)	15,45 ± 6,25 (6,38 – 35,00)
Group C (*n* = 34)	69,50 ± 9,52 (54,20 – 91,80)	103,98 ± 6,32 (91,20 – 120,00)	141,97 ± 12,45 (125,00 – 179,00)	15,89 ± 5,46 (7,96 – 31,30)
Group D (*n* = 32)	71,07 ± 13,93 (47,20 – 96,50)	105,02 ± 7,48 (91,80 – 119,00)	144,41 ± 13,47 (109,00 – 181,00)	13,22 ± 4,86 (4,40 – 23,80)

The P100 latency was significantly larger the older the test person was (F(2,135) = 6.162, *p* = 0.003). The amplitude (N75-P100) was significantly smaller in elderly test subjects (F(2,135) = 15.558, *p* < 0.001). The P100 latency was significantly shorter in women (*p* = 0.003), and the amplitude (N75-P100) was significantly greater in female study participants (*p* < 0.001). For the N75 component of the PR- FF-VEP, age and gender did not cause significant changes although the tendency was the same as for the P100. N135 values showed no significant effect of age and sex (see Table 4).

**Table 4:** Multiple regression analysis for phakic eyes for the fullfield pattern-reversal VEP (FF-PR-VEP) with 1.4° ss;

Target variable	ANOVA (*p*-value)	Significance of coefficient		Regression coefficient B	
		Age	Sex	Age	Sex
N75 [ms]	0,520	0,338	0,487	0,037	− 1,230
P100 [ms]	0,003*	0,016*	0,020*	0,071*	− 2,844*
N135 [ms]	0,110	0,163	0,096	− 0,091	− 4,521
Ln(N75-P100) [μV]	< 0,001*	0,011*	< 0,001*	− 0,004* (0,996)	0,292* (1,339)

Results of ANOVA, the significance of coefficient and the regression coefficient B; transformation by natural logarithm (ln); data in brackets refer to back transformed values; level of significance is 5% (*); *n* = 138

When stimulating with small checks (ss 20.4′), mean P100 latency was 103.81 ± 7.77 ms (84.20–124.00) for the phakic eyes and the mean amplitude was 16.30 ± 7.53 µV (5.07–44.30 µV) (see Table 5).

**Table 5:** Reference values (mean value ± standard deviation (range)) for latencies (N75, P100, N135) and amplitude (N75-P100) of phakic test persons for the fullfield pattern-reversal VEP (FF-PR-VEP) with 20.4′ ss

	N75 [ms]	P100 [ms]	N135 [ms]	N75-P100 [μV]
Total (*n* = 138)	78,09 ± 7,30 (55,40–98,30)	103,81 ± 7,77 (84,20–124,00)	144,95 ± 12,78 (112,00–185,00)	16,30 ± 7,53 (5,07–44,30)
Group A (*n* = 28)	74,93 ± 6,35 (62,40–84,80)	101,50 ± 8,43 (84,20–122,00)	138,21 ± 11,21 (112,00–156,00)	17,19 ± 8,82 (6,14–44,30)
Group B (*n* = 44)	75,99 ± 7,33 (55,40–87,10)	101,54 ± 6,21 (91,80–120,00)	145,14 ± 12,13 (122,00–185,00)	15,57 ± 8,30 (5,07–41,40)
Group C (*n* = 34)	79,19 ± 5,73 (64,80–88,90)	103,96 ± 7,78 (90,00–124,00)	144,97 ± 14,39 (123,00–181,00)	17,12 ± 5,77 (7,08–31,50)
Group D (*n* = 32)	82,58 ± 7,30 (67,70–98,30)	108,78 ± 7,05 (93,00–121,00)	150,56 ± 10,69 (135,00–176,00)	15,65 ± 7,00 (5,65–31,40)

N75, P100 and N135 were significantly larger the older the test person was (N75: F(2,135) = 13.32, *p* < 0.001, P100: F(2,135) = 11.16, *p* < 0.001, N135: F(2;135) = 6.849, *p* = 0.001). There was no significant difference concerning the amplitude when referring to the age of the test person. The amplitude was significantly larger in women compared to men (*t* = 6.247, *p* < 0.001) (see Table 6).

**Table 6:** Multiple regression analysis for phakic eyes for the fullfield pattern-reversal VEP (FF-PR-VEP) with 20.4′ ss;

Target variable	ANOVA (*p*-value)	Significance of coefficient		Regression coefficient B	
		Age	Sex	Age	Sex
N75 [ms] FF 20.4′	< 0,001*	< 0,001*	0,911	0,144*	− 0,129
P100 [ms] FF 20.4′	< 0,001*	< 0,001*	0,965	0,142*	− 0,055
Ln(N135) [ms] FF 20.4′	0,001*	< 0,001*	0,337	0,001* (1,001)	0,014 (1,014)
Ln(N75-P100) [μV] FF 20.4′	< 0,001*	0,749	< 0,001*	0,001 (1,001)	0,430* (1,537)

Results of ANOVA, the significance of coefficient and the regression coefficient B; transformation by natural logarithm (ln); data in brackets refer to back transformed values; level of significance is 5% (*); *n* = 138

The subdivision of group D in phakic and pseudophakic eyes showed no significant difference for N75, P100, N135 and N75-P100 when stimulating with large checks (ss 1.4°) (N75: *t*(54) = 0.724, *p* = 0.472, P100: *t*(54) = − 0.158, *p* = 0.875, N135: *t*(54) = − 1.140, *p* = 0.259, N75-P100: *t*(54) = − 0.561, *p* = 0.577) and also when stimulating with small checks (ss 20.4′) (N75: t(65) = − 0.654, *p* = 0.516, P100: *t*(54) = − 1.027, *p* = 0.309, N135: *t*(54) = − 0.923, *p* = 0.363, N75-P100: *t*(54) = − 1.070, *p* = 0.290) (see Tables 7 and 8).

**Table 7:** Reference values (mean value ± standard deviation (range)) for latencies (N75, P100, N135) and amplitude (N75-P100) of phakic and pseudophakic test persons of group D for the fullfield pattern-reversal VEP (FF-PR-VEP) with 1.4° ss

Target variable	N75 [ms]	P100 [ms]	N135 [ms]	N75-P100 [μV]
Phakic (*n* = 32)	71,07 ± 13,93 (47,20–96,50)	105, 02 ± 7,48 (91,80–119,00)	144,41 ± 13,47 (109,00–181,00)	13,22 ± 4,86 (4,40–23,80)
Pseudophakic (*n* = 24)	68,38 ± 13,45 (51,30–96,50)	105,37 ± 9,33 (83,60–119,00)	149,25 ± 18,36 (121,00–203,00)	13,98 ± 5,16 (6,89–23,50)

**Table 8:** Reference values (mean value ± standard deviation (range)) for latencies (N75, P100, N135) and amplitude (N75-P100) of phakic and pseudophakic test persons of group D for the fullfield pattern-reversal VEP (FF-PR-VEP) with 20.4° stimulus size (ss)

Target variable	N75 [ms]	P100 [ms]	N135 [ms]	N75-P100 [μV]
Phakic (*n* = 32)	82,58 ± 7,30 (67,70–98,30)	108,78 ± 7,05 (93,00–121,00)	150,56 ± 10,69 (135,00–176,00)	15,65 ± 7,00 (5,65–31,40)
Pseudophakic (*n* = 24)	83,86 ± 7,20 (68,90–99,40)	110,89 ± 8,33 (98,80–128,00)	154,96 ± 21,41 (126,00–227,00)	17,69 ± 7,18 (7,56–36,20)

### Double-peaked VEP

**Fig. 4:**
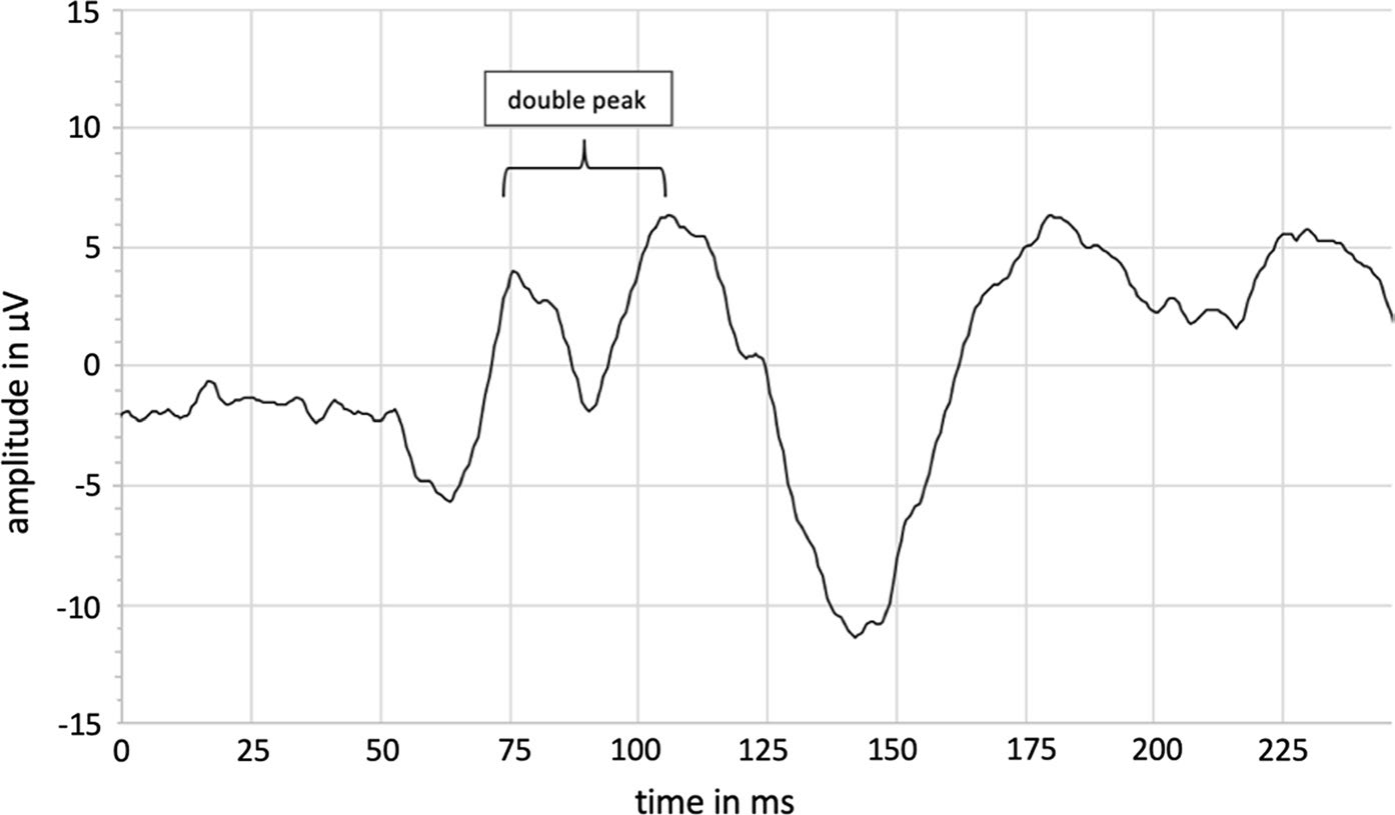
Fullfield pattern-reversal VEP (FF-PR-VEP) with double-peaked configuration of a female test subject in group D (ss 20.4′)

**Fig. 5:**
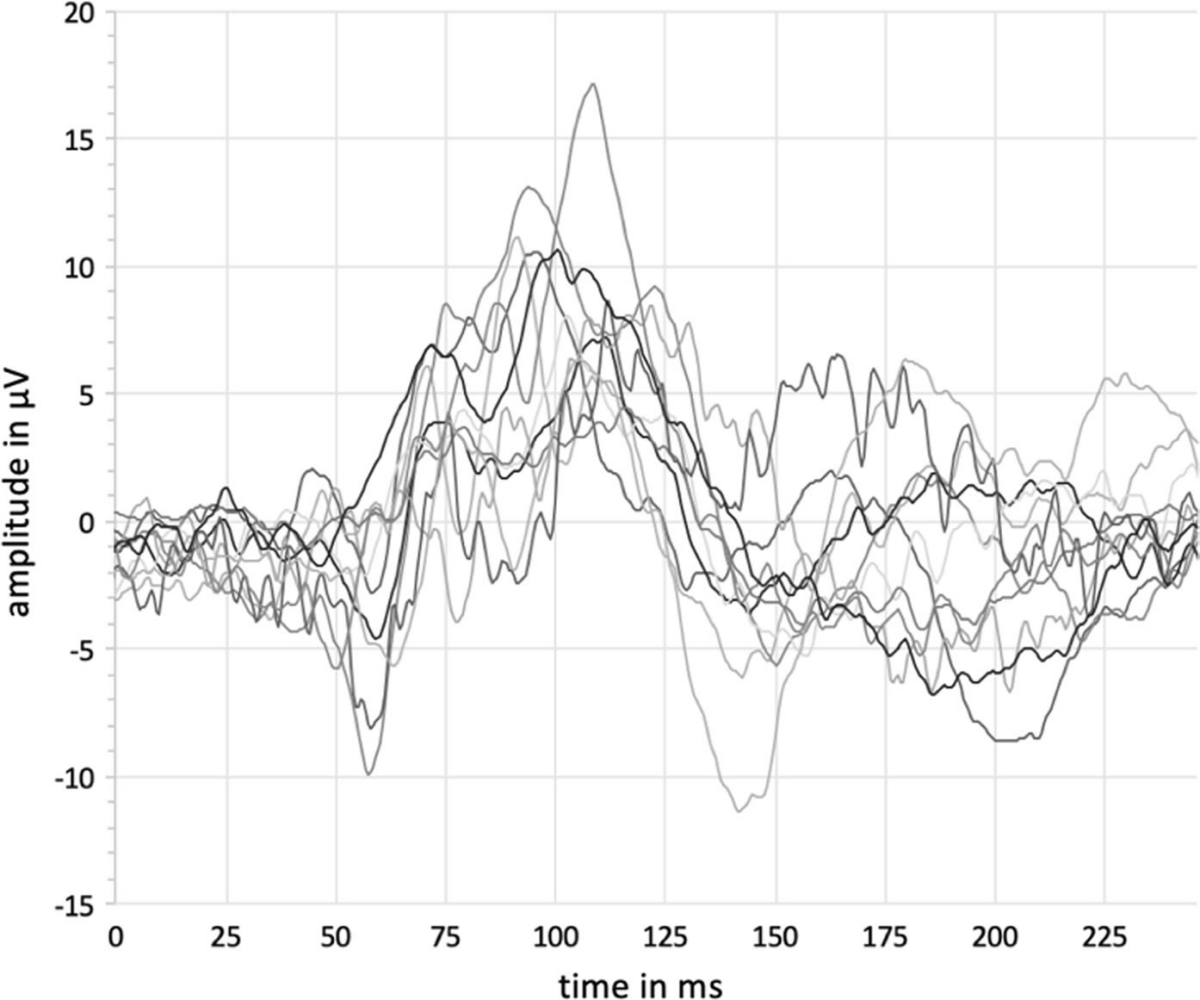
Fullfield pattern-reversal VEP (FF-PR-VEP) waveforms (*n* = 11) with double-peak configuration of phakic and pseudophakic test subject in group D (stimulus size (ss) 20.4′)

In group D, in the FF-PR-VEP (ss 20.4′), 11 double-peaked P100 wave configurations (see Fig. 4, Fig. 5 and Table 9) were seen (15.6% (5/32) of phakic eyes and 25.0% (6/24) of pseudophakic eyes).

**Table 9:** Mean value ± standard deviation (range)) for latencies (N75, P100 1. peak and P100 2. peak) of phakic and pseudophakic test persons (*n* = 11) with double-peak configurations for the fullfield pattern-reversal VEP (FF-PR-VEP) with 20.4′ stimulus size (ss)

	N75 [ms]	P100 [ms] 1. peak	P100 [ms] 2.peak
Total (*n* = 11)	68.89 ± 16.95 (47.20–94.10)	77.85 ± 5.66 (70.40–88.60)	105.73 ± 8.87 (91.8–118.00)

### Extrafoveal pattern onset–offset VEP (EF-P-ON/OFF-VEP)

Data for the EF-P-ON/OFF-VEP of phakic eyes for both check sizes (ss 2.4° and 2.8°) are given in Tables 10 and 11:

**Table 10:** Reference values (mean value ± standard deviation (range)) for latencies (C1, C2, C3) and amplitude (C1–C2) of phakic test persons for the extrafoveal pattern onset/offset VEP (EF-P-ON/OFF-VEP) with 1.4° ss

	C1 [ms]	C2 [ms]	C3 [ms]	C1–C2 [μV]
Total (*n* = 138)	77,18 ± 8,34 (56,00–115,00)	102,95 ± 11,84 (73,60–131,00)	134,72 ± 21,99 (89,40–217,00)	13,48 ± 9,29 (0,02–63,60)
Group A (*n* = 28)	74,91 ± 10,68 (61,30–115,00)	94,79 ± 12,00 (73,60–126,00)	121,35 ± 20,24 (89,40–171,00)	7,58 ± 6,86 (0,02–25,60)
Group B (*n* = 44)	73,65 ± 7,14 (56,00–90,60)	98,02 ± 8,45 (74,80–121,00)	126,00 ± 20,11 (101,00–176,00)	9,13 ± 5,42 (0,95–22,70)
Group C (*n* = 34)	78,68 ± 6,61 (63,00–94,10)	106,48 ± 8,42 (88,90–122,00)	140,88 ± 12,61 (111,00–174,00)	19,07 ± 10,69 (5,40–63,60)
Group D (*n* = 32)	82,43 ± 6,12 (71,80–94,10)	113,10 ± 10,21 (93,00–131,00)	151,84 ± 20,87 (107,00–217,00)	18,70 ± 7,54 (7,15–38,00)

**Table 11:** Reference values (mean value ± standard deviation (range)) for latencies (C1, C2, C3) and amplitude (C1–C2) of phakic test persons for the extrafoveal pattern onset/offset VEP (EF-P-ON/OFF-VEP) with 2.8° ss

	C1 [ms]	C2 [ms]	C3 [ms]	C1–C2 [μV]
Total (*n* = 138)	77,76 ± 6,71 (59,50 – 94,10)	102,79 ± 12,41 (71,80 – 131,00)	131,67 ± 23,96 (88,90 – 213,00)	12,43 ± 9,15 (0,01 – 63,20)
Group A (*n* = 28)	76,06 ± 6,52 (64,80 – 90,60)	93,73 ± 10,83 (71,80 – 120,00)	114,61 ± 18,23 (88,90 – 169,00)	6,45 ± 5,58 (0,01 – 21,70)
Group B (*n* = 44)	76,32 ± 5,66 (66,00 – 88,90)	97,07 ± 7,43 (81,80 – 113,00)	120,30 ± 18,48 (98,30 – 181,00)	8,04 ± 5,06 (0,34 – 19,00)
Group C (*n* = 34)	78,27 ± 6,85 (59,50 – 93,00)	107,51 ± 11,45 (75,90 – 131,00)	140,03 ± 17,38 (106,00 – 179,00)	17,13 ± 10,98 (2,61 – 63,20)
Group D (*n* = 32)	80,68 ± 7,27 (61,30 – 94,10)	113,58 ± 9,87 (93,00 – 129,00)	153,34 ± 21,06 (107,00 – 213,00)	18,69 ± 7,41 (7,93 – 37,50)

In the EF-P-ON/OFF-VEP, C1, C2 and C3 were significantly larger the older the test subject was (C1: F(2,135) = 12.886, *p* < 0.001, C2: F(2,135) = 39.840, *p* < 0.001, C3: F(2,135) = 32.730, *p* < 0.001). There were no gender-specific significant differences. These two latter aspects apply to both ss (1.4° and 2.8°). The amplitude (C1-C2) was significantly greater the older the test subject was (F(2,135) = 39.423, *p* < 0.001), and the amplitude was again significantly greater in women (see Tables 12 and 13).

**Table 12:** Multiple regression analysis for phakic eyes for the extrafoveal pattern onset/offset VEP (EF-P-ON/OFF-VEP) with 1.4° ss;

Target value	ANOVA (*p*-value)	Significance of coefficient		Regression coefficient B	
		Age	Sex	Age	Sex
C1 [ms]	< 0,001*	< 0,001*	0,612	0,162*	0,672
C2 [ms]	< 0,001*	< 0,001*	0,820	0,349*	− 0,370
C3 [ms]	< 0,001*	< 0,001*	0,837	0,609*	− 0,644
C1-C2 [μV]	< 0,001*	< 0,001*	< 0,001*	0,255*	4,892*

Results of ANOVA, the significance of coefficient and the regression coefficient B; transformation by natural logarithm (ln); level of significance is 5% (*); *n* = 138

**Table 13:** Multiple regression analysis for phakic eyes for the extrafoveal pattern onset/offset VEP (EF-ON/OFF-VEP) with 2.8° ss;

Target value	ANOVA (*p*-value)	Significance of coefficient		Regression coefficient B	
		Age	Sex	Age	Sex
C1 [ms]	0,006*	0,002*	0,520	0,085*	− 0,723
C2 [ms]	< 0,001*	< 0,001*	0,767	0,387*	− 0,489
C3 [ms]	< 0,001*	< 0,001*	0,950	0,779*	0,193
Ln(C1–C2) [μV]	< 0,001*	< 0,001*	< 0,001*	0,030* (1,030)	0,561* (1,752)

Results of ANOVA, the significance of coefficient and the regression coefficient B; transformation by natural logarithm (ln); data in brackets refer to back transformed values; level of significance is 5% (*); *n* = 138

For both ss (1.4° and 2.8°), there were no statistic significant differences in the EF-P-ON/OFF-VEP between phakic and pseudophakic eyes in C1, C2 and C3 and C1-C2.

In the subanalysis (*n* = 10) of EF-P-ON/OFF-VEP versus FF-P-ON/OFF-VEP, for ss 1.4°, there were no significant differences found for C1, C2 and C3 (C1: W = 19.50, *p* = 0.449, C2: *W* = 19.00, *p* = 0.432, C3: *W* = 12.00, *p* = 0.238). The amplitude C1–C2 was significantly greater after FF stimulation compared to EF (W = 7.50, *p* = 0.041). Effect size according to the classification by Cohen was strong with *r* = 0.64.

When stimulating with ss 2.8°, all parameters (C1, C2, C3, C1–C2) showed no significant differences (C1: *W* = 18.00, *p* = 0.375, C2: *W* = 19.00, *p* = 0.434, C3: *W* = 18.00 *p* = 0.375, C1–C2: *W* = 25.00, *p* = 0.846).

## Discussion

In this study we established age-related reference values for the FF-PR- and EF-P-ON/OFF-VEPs in healthy eyes for our laboratory and examined age- and gender-specific influences on latencies and amplitudes.

### Influence of age on latency

We found age-dependent significant differences for the P100 latency in the FF-PR-VEP for both ss (20.4′ and 1.4°). In group A, the P100 latency was greater (mean P100 = 102.13 ± 8.29 ms) than in group B (mean P100 = 100.01 ± 6.41 ms) for 1.4° ss and in group C and D, P100 latency was increasing again (group C P100 = 103.98 ± 6.32 ms, group D P100 = 105.02 ± 7.48 ms). So between twenty and 39 years, P100 latency was the shortest.

Significant slowing of the P100 latency in elderly persons has been demonstrated in previous reports [20–23]. The recent study by Benedek et al. supported most of the findings in the literature concerning the aging of VEP components [22]: They showed that latencies of P100 and N135 decrease up to the third decade of life and then show an increase again. In our study, we saw an average increase in P100 latency of 0.07 ms per year in the FF-PR-VEP. In the EF-P-ON/OFF-VEP, a statistically significant increase in C1-, C2-, C3-latency was also seen. Benedek et al. hypothesized that the P100 latency is the most sensitive component to age and many studies confirm this hypothesis [8, 24–27]. There are reports that the N75 latency is also affected by aging processes, however, in a different way compared to the P100 latency: N75 is being modulated linearly and P100 curvilineary with U-shaped configurations [10, 11, 28]. In our study, there was no statistic significant difference in N75 concerning age when stimulating with large checks (ss 1.4°).

However, when stimulating with smaller checks (ss 20.4′), we observed a statistic significant increase in N75, P100 and N135 latency (*p* < 0.001). So our data confirm the importance of check size also in studying aging effects as reported before in the literature. Sokol et al. showed that the rate of latency increases with age twice as fast for checks of 12′ than for checks of 48′ [9].

### Influence of age on amplitude

N75-P100 amplitude was also modulated by age: In the FF-PR-VEP, a significant decrease in N75-P100 amplitude of 0.4% per year was observed when stimulating with large checks (ss 1.4°) and no statistically significant age-related change was seen when stimulating with smaller checks (ss 20.4′). In the EF-P-ON/OFF-VEP, C1-C2 amplitude increased statistically significant each year for both stimulus sizes (ss 1.4° and 2.8°). In the literature, there also exist miscellaneous data concerning age-related amplitude changes. Shaw et al. reported in 1981 P100 amplitudes being the greatest in childhood, then declining until the forth decade, increasing again and after the sixth decade, a decrease of P100 amplitude is again observed [12]. Tobimatsu et al. observed no aging effect on P100 amplitude; here PR-VEPs were recorded in 109 normal subjects with different stimulus conditions (inter alia high versus low luminance or different check sizes) [29]. Their results suggested that age-related changes in the human visual system are not uniform, but rather different in the specific functional subdivisions [29]. They hypothesized that aging may differentially influence the separate channels of human visual system [29]. This is also true for our study as different retinal stimulus localizations (EF vs FF) caused different age-related VEP changes.

### Influence of sex on latency and amplitude

Considering the influence of sex on FF-PR-VEP components (ss 1.4°) we observed the following: The P100 latency was on average 2.84 ms shorter in female test persons compared to male subjects of the same age. Furthermore, the N75-P100 amplitude was on average 33.9% greater in women compared to men. When stimulating with 20.4°, the N75-P100 amplitude was on average 53.7% greater in females compared to males (*p* < 0.001) and in the EF-P-ON/OFF-VEP, the C1–C2 amplitude was again significantly greater in women compared to men for both ss (1.4° and 2.8°, *p* < 0.001). There seems to be general agreement in the literature that sex has a significant influence on VEP components: With different check sizes various authors reported on greater N75-P100 amplitudes and shorter P100 latencies in women compared to men [11, 13, 14, 30–33]. The exact reason of these gender differences in VEP parameters is not totally understood, but it may be associated with endocrinal, anatomical and behavioral differences.

Some investigators associated a shorter head circumference with a shorter VEP latency: Guthkelch et al. reported on eight male and eight female healthy young adults showing the shortest latency to P100 when having the lowest occipito-frontal circumference (OFC) [32]. This variation in P100 latency was even more highly correlated with OFC than with gender: Men with the same OFC as women showed comparable latencies [32]. A more recent study including 400 eyes showed a positive correlation of P100 latency with mean head circumference, while a highly significant negative correlation was observed of N75-P100 amplitude with head circumference [34]. They concluded that a larger head circumferences indicate a larger brain size and a longer conduction pathway, thus prolonging VEP latencies [34].

### Influence of lens status

Regardless of ss there was no statistic significant difference to be seen between phakic and pseudophakic eyes in the FF-PR-VEP as well as in the EF-P-ON/OFF-VEP for latencies and amplitudes. In the literature different studies report that the senile opacity of the crystalline lens does not contribute to changes of PR-VEPs [37, 38]. Yamamoto et al. assumed that longer latencies in elder test subjects are due to general senile changes in the optic pathway and not due to reduced transparency of the crystalline lens in elderly [37].

### Double-peaked VEP

A particular phenomenon that we noticed in patients older than sixty for the P100 response in the FF-PR-VEP was several double-peaked P100 wave configurations. In the literature we found that this phenomenon is sometimes considered as a sign of demyelization [39] but can also be found in healthy older adults [40].

### Comparison to VEP reference values with other display types

We compared our VEP reference values with those reported by Ekayanti et al.[41]. They used a 24-in DELL LCD monitor (Dell, Round Rock, USA) for their pattern-reversal VEP examination in 120 healthy subjects between 18 and 65 years. Their reference values were slightly slower than ours in each age group (approx. 1–3 ms). They did not see significant differences between the P100 latency values of gender and age groups [41]. However, as in our study, the P100 amplitude was significantly higher in females compared to males [41]. It is known that LCD displays cause apparent (artificial) delay in the P100 latency due to the input lag of the monitor [42]. There are some software modifications that can compensate for the lag [43]. The study by Husain et al. [42] confirmed the assumption that substituting a cathode-ray tube (CRT) monitor with a LCD monitor results in a significant prolongation of P100 latency. CRT monitors have become less available in the market and liquid crystal displays (LCD) [16] have an inherent problem as visual stimulators that is called “the flash effect” [44]. LCDs take several milliseconds for the crystal molecules to change their alignment to permit the light to pass through the polarizing filter of the LCD [16, 45], and this causes a transient change of the mean luminance of the entire LCD screen at the time of the reversal and this luminance change can elicit flash VEPs; that’s where the “flash effect” comes from [44]. So the question is how the recently developed OLED monitor influences VEP values and whether this monitor is suitable for eliciting VEPs. Here we think that the work published by Matsumoto et al. is very important [16] as they examined whether OLED screens can be used as visual stimulators. They showed that p-VEPs elicited by OLED screens were not significantly different from those elicited by conventional CRT screens [16, 44] as OLED screens have a faster response time than standard LCD screens [46, 47]. They showed that OLED displays are suitable for a visual stimulator to elicit p-VEPs.

Other authors explored the characteristics of OLED displays for its applicability in visual research [48, 49]. They found the new display to be superior to other display types in terms of spatial uniformity, color gamut and contrast ratio [48]. However, there are no studies on VEP data when a curved OLED monitor was used and this is new in our study.

### Extrafoveal stimulation

The contribution of the peripheral retina to pattern VEP is a matter of debate. Some authors stated that the VEP is primarily a reflection of activity originating in the central two to six degrees of the visual field [50] so the central macular response dominates the VEP response [34, 51–53]. It had been known for nearly a century now that each visual area has a retinotopic organization in human striate cortex. Meredith and Celesia reported in 16 healthy volunteers on an amplitude distribution of evoked responses in relation to retinal eccentricity [54] and confirmed previous research, namely that the amplitude distribution correlates well with (1) the decline in cone density in relation to retinal eccentricity [55], (2) the density distribution of human ganglion cell population along the horizontal axis [56] and (3) the decline of visual acuity in relation to eccentricity [57, 58]. These correlations further suggest that a visual stimulus outside the fovea has to reach the threshold of visual perception to be effective and needs to activate sufficient numbers of receptors and ganglion cells [54].

Walter and colleagues compared amplitudes in PR-VEPs in patients with age-related macular degeneration (AMD) and found a few individuals showing larger amplitudes after stimulation of a central 3° field compared to stimulation of a 13° field although in normals and the majority of AMD patients, VEP amplitudes increased with increasing field size [59]. After stimulation of different macular zones they found that the 3° central area and the perifoveal region contributed differently to the macular response or 13° response [59]. They suggested that cortical magnification factor in AMD might be higher than in normal controls [59].

In our study, in the FF-P-ON/OFF-VEP (ss 1.4°), amplitudes were significantly greater to amplitudes in the EF-P-ON/OFF-VEP (*p* = 0.041), but there were no statistically significant differences for the C1-, C2, and C3-latencies (*p* = 0.238). However, in this subanalysis only ten test persons were included so the validity of this subanalysis is limited. Comparing our reference values of the EF-P-ON/OFF-VEP with data on FF-P-ON/OFF-VEPs by Thompson and colleagues on 24 healthy adult test persons, the C1-, C2- and C3-latencies of our extrafoveal VEP were shorter [60]. Hagler [61] confirmed significantly shorter latencies after peripheral compared to perifoveal stimulation in PR-VEP, and we did expect this tendency of shorter latencies when occluding the central retina due to the differences in axonal conduction speed between the magnocellular and parvocellular pathways.

In order to explain the contribution of the peripheral retina to VEP components the anatomical composition of neural macro-networks that process the visual information has to be considered. Starting in non-human primates much about the two major parallel retinocortical pathways, the magnocellular (M) and the parvocellular (P) pathway, has been described [62–64]: The M pathway begins with the parasol ganglion cells of the retina [65]. These cells have large receptive fields and selectively project to the magnocellular layers of the lateral geniculate nucleus (LGN) [65]. Midget cells of the retina are the origin of the P pathway, representing approximately eighty percent of ganglion cells [65]. Midget cells have small dendritic and receptive fields and project mainly to the parvocellular layers of the LGN [65]. Both pathways project in different layers of the primary visual cortex (V1) to become dorsal (M) and ventral (P) streams [66].

Concerning physiological aspects, M cells of the retina and the LGN are relatively insensitive to pure chromatic contrast, but highly sensitive to luminance contrast [65, 67]. P cells are sensitive to chromatic contrast, but less sensitive to luminance contrast compared to M cells [64, 65, 68]. The M pathway is sensitive to lower spatial frequencies and higher temporal frequencies and has transient responses [69]. The P pathway is responsive to higher spatial frequencies and lower temporal frequencies and sustained responses [69–71].

The relative distributions of neurons within the M and P pathway are still discussed [65]. A study in macaque LGN by Malpeli et al. estimated the magnocellular/parvocellular ratio to increase from the foveolar to far periphery by a factor of at least 14 [72]. More recent studies compared the magno- and parvocellular distributions in the human retina and found a decrease in parvocellular/magnocellular ratio with eccentricity which was even more distinctive than that of macques [73, 74]. There exist several studies that report on the differences in the relative speed of the magnocellular and parvocellular pathways: Differences in axonal conduction speeds in the retina, optic nerve and optic radiation are expected to cause parvocellular signals approximately 3 ms longer than magnocellular signals to travel to the LGN and about 5 ms longer to get to the cerebral cortex [75–77]. In our study, for the EF-P-ON/OFF-VEP, we chose larger ss as for the FF-PR-VEP (1.4° und 2.8° compared to 20.4′ and 1.4°) to favor the lower resolution of the M pathway.

The EF approach in our study has an experimental character, and with this analysis, we aim to pave the way for new VEP testing modalities that allow us to use VEPs effectively for example in the diagnosis and management of glaucoma.

So all in all, with this study we determined reference values with the OLED monitor LG55EC930V for the FF-PR-VEP and EF-PR-ON/OFF-VEP in healthy test persons and investigated gender and age-related influences. No impact of lens status was seen and extrafoveal deductions showed the tendency to have smaller amplitudes and shorter latencies compared to FF deductions.
